# Supplementary material for: Structure of a tetrameric photosystem I from a glaucophyte alga Cyanophora paradoxa
Source: Nat Commun. 2022 Mar 30;13:1679. doi: 10.1038/s41467-022-29303-7 (PMC8967866; doi:10.1038/s41467-022-29303-7)
Supplement: Supplementary file 1 — Supplementary Information [file 41467_2022_29303_MOESM1_ESM.pdf]

Supplementary Information

Structure of a tetrameric photosystem I from a glaucophyte alga *Cyanophora paradoxa*

Koji Kato[1,†], Ryo Nagao[1,†,*], Yoshifumi Ueno[2], Makio Yokono[3], Takehiro Suzuki[4], Tian-Yi Jiang[1], Naoshi Dohmae[4], Fusamichi Akita[1], Seiji Akimoto[2], Naoyuki Miyazaki[5,*], and Jian-Ren Shen[1,*]

[1]Research Institute for Interdisciplinary Science and Graduate School of Natural Science and Technology, Okayama University, Okayama 700-8530, Japan
[2]Graduate School of Science, Kobe University, Hyogo 657-8501, Japan
[3]Institute of Low Temperature Science, Hokkaido University, Hokkaido 060-0819, Japan
[4]Biomolecular Characterization Unit, RIKEN Center for Sustainable Resource Science, Saitama 351-0198, Japan
[5]Life Science Center for Survival Dynamics, Tsukuba Advanced Research Alliance (TARA), University of Tsukuba, Ibaraki 305-8577, Japan
[†]These authors contributed equally to this work.

*Corresponding Authors:
Ryo Nagao, E-mail: nagaoryo@okayama-u.ac.jp
Naoyuki Miyazaki, E-mail: naomiyazaki@gmail.com
Jian-Ren Shen, E-mail: shen@cc.okayama-u.ac.jp

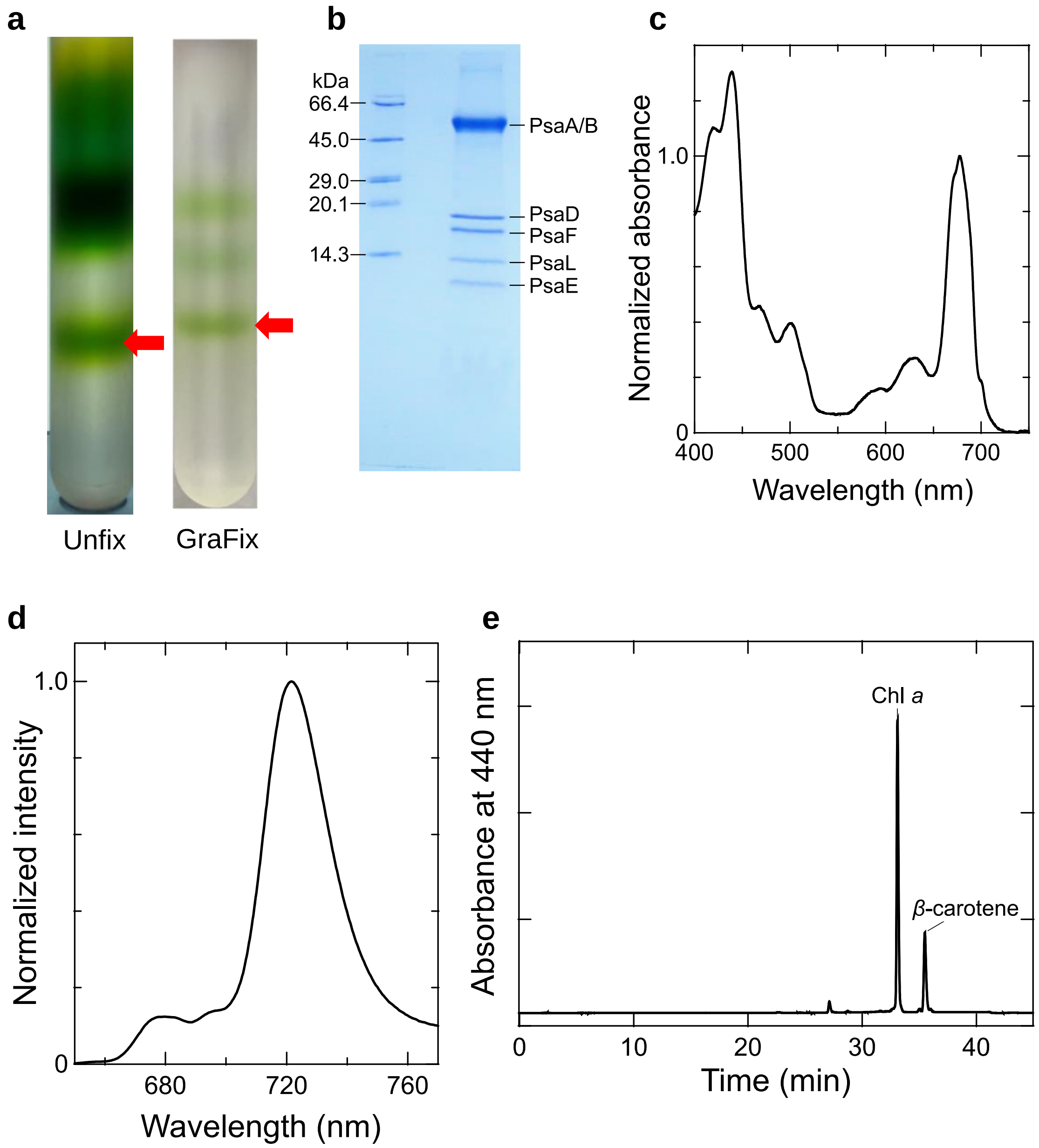

Supplementary Fig. 1. Biochemical and spectroscopic characterizations of the PSI tetramer. a, Trehalose gradient centrifugation of the unfix (left) and GraFix (right) PSI preparations from *Cyanophora paradoxa.* Red arrows indicate PSI tetramer. **b,** SDS-PAGE analysis of the PSI tetramer obtained from trehalose gradient centrifugation shown in panel **a**. **c,** Absorption spectrum of the PSI tetramer measured at 77 K. **d,** Normalized fluorescence spectrum of the PSI tetramer excited at 445 nm and measured at 77 K. **e,** HPLC analysis of pigments extracted from the PSI tetramer monitored at 440 nm. Data are representative of two or three independent experiments.

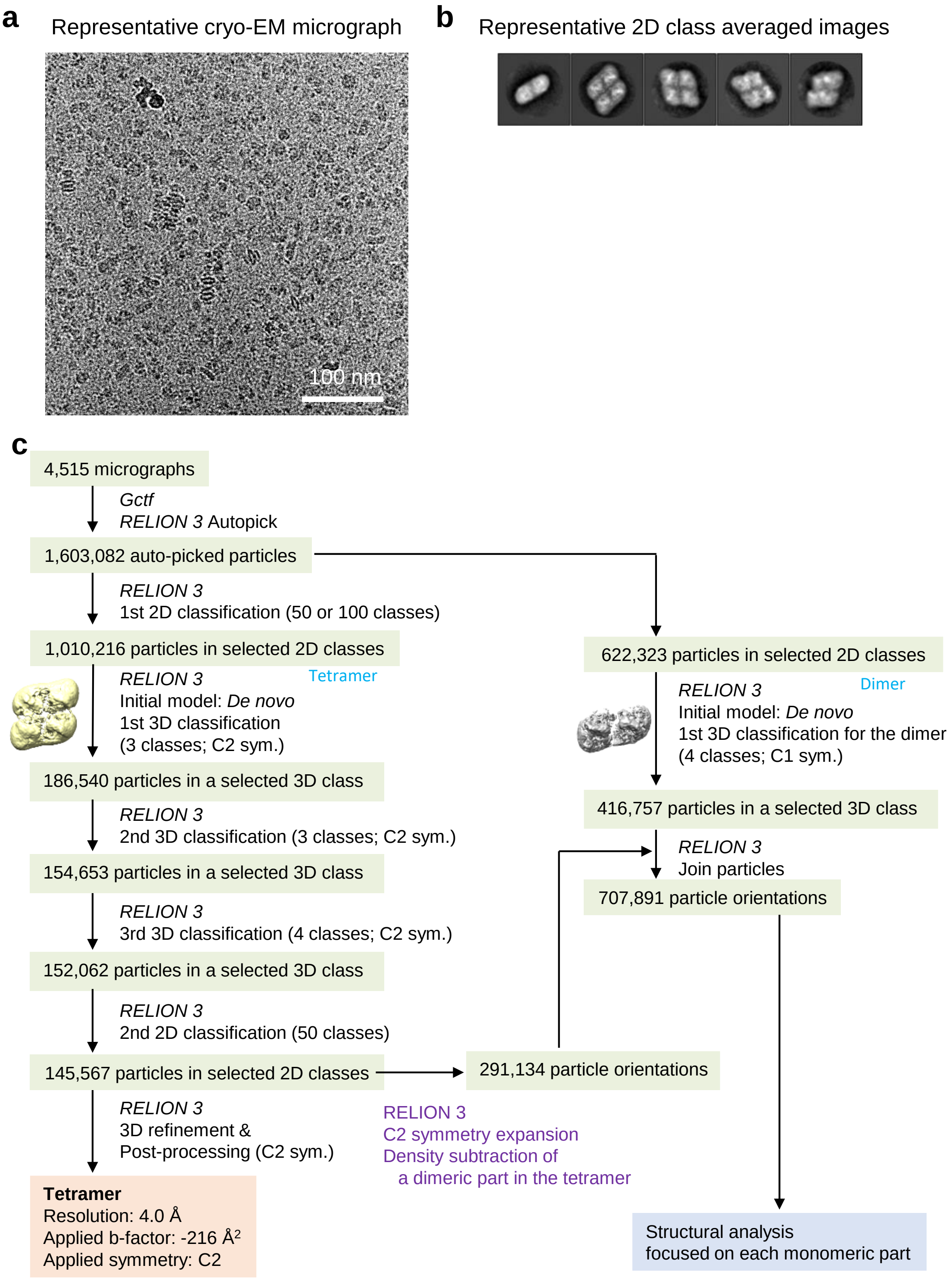
a
Representative cryo-EM micrograph
100 nm
b
Representative 2D class averaged images
c
4,515 micrographs
Gctf
RELION 3 Autopick
1,603,082 auto-picked particles
RELION 3
1st 2D classification (50 or 100 classes)
1,010,216 particles in selected 2D classes
RELION 3
Tetramer
Initial model: De novo
1st 3D classification
(3 classes; C2 sym.)
186,540 particles in a selected 3D class
RELION 3
2nd 3D classification (3 classes; C2 sym.)
154,653 particles in a selected 3D class
RELION 3
3rd 3D classification (4 classes; C2 sym.)
152,062 particles in a selected 3D class
RELION 3
2nd 2D classification (50 classes)
145,567 particles in selected 2D classes
RELION 3
3D refinement &
Post-processing (C2 sym.)
Tetramer
Resolution: 4.0 Å
Applied b-factor: -216 Å2
Applied symmetry: C2
622,323 particles in selected 2D classes
RELION 3
Dimer
Initial model: De novo
1st 3D classification for the dimer
(4 classes; C1 sym.)
416,757 particles in a selected 3D class
RELION 3
Join particles
707,891 particle orientations
291,134 particle orientations
RELION 3
C2 symmetry expansion
Density subtraction of
a dimeric part in the tetramer
Structural analysis
focused on each monomeric part

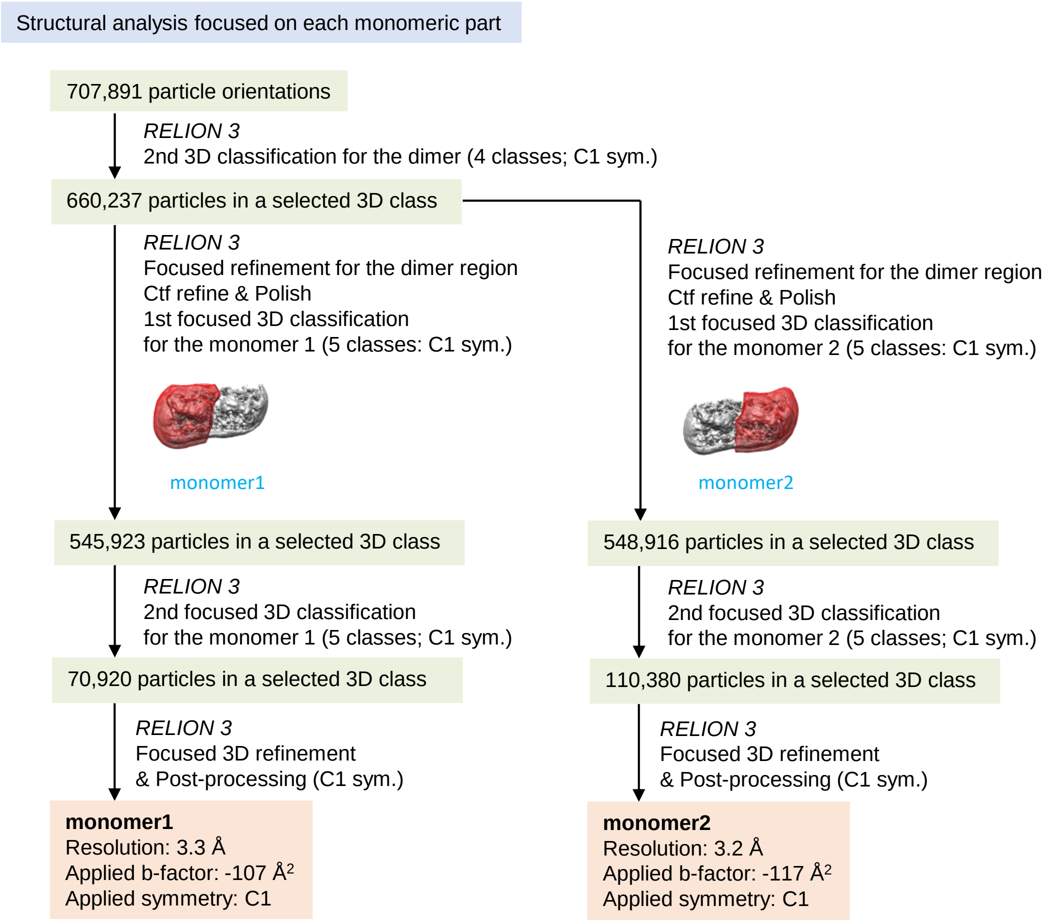

Supplementary Fig. 2. Cryo-EM data collection and processing of the wild-type PSI tetramer. a, Representative cryo-EM micrograph of the PSI tetramer. **b,** Representative 2D classes of the PSI tetramer. The box size is 437 Å. **c,** Schematic flowchart showing the classification scheme and data processing for the PSI tetramer. The overall PSI-tetramer structure was reconstructed at 4.0 Å resolution from 145,567 particles, whereas monomer1 and monomer2 structures from the subtracted particles were reconstructed at resolutions of 3.3 and 3.2 Å, respectively. See Methods section for more details.

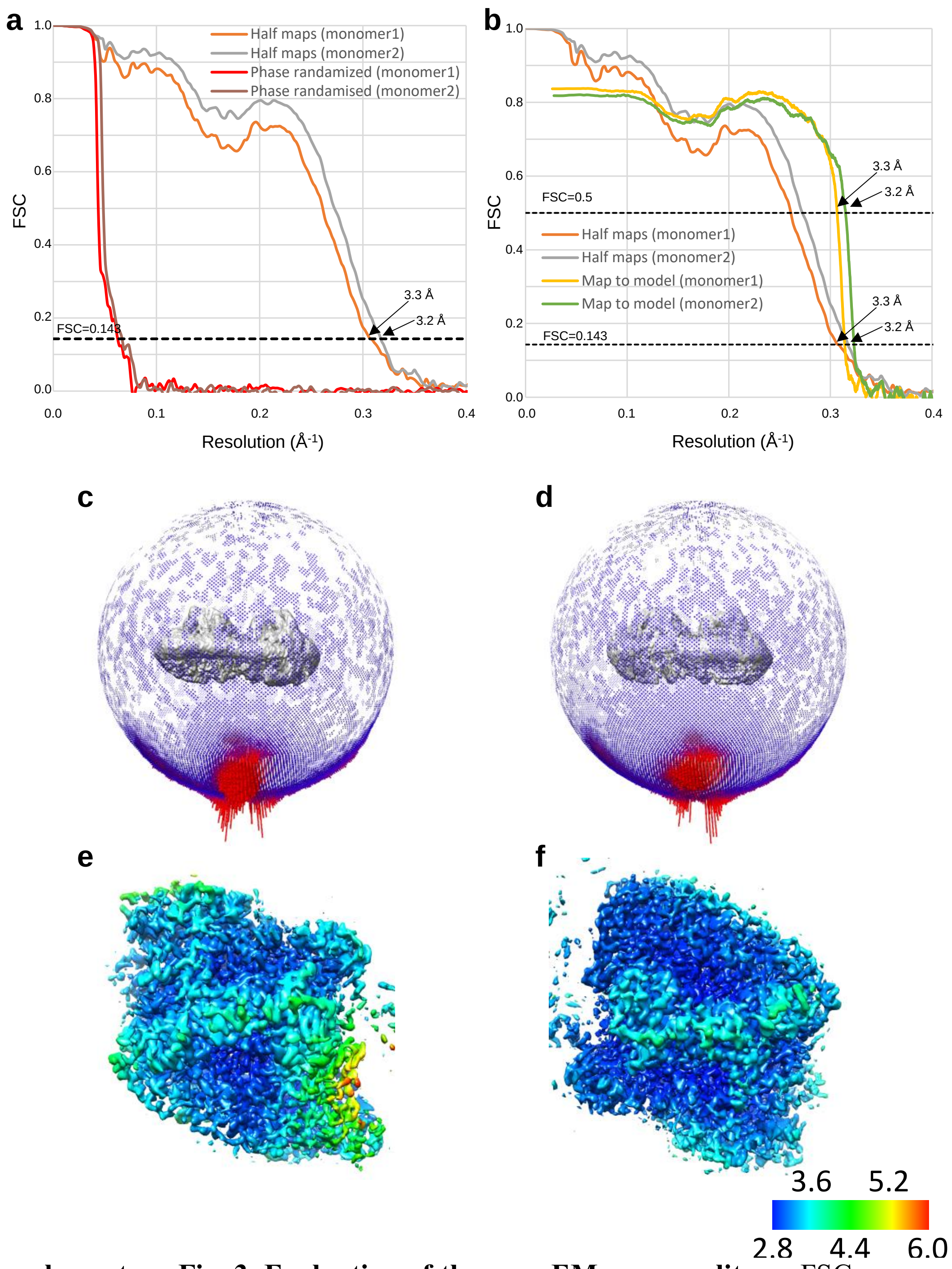

Supplementary Fig. 3. Evaluation of the cryo-EM map quality. a, FSC curves of the wild-type monomer1 and monomer2 for independently refined half-maps (half-maps) and phase randomized maps. **b,** FSC curves of the wild-type monomer1 and monomer2 for independently refined half-maps (half-maps) and full map vs. model (map to model). **c, d,** Angular distributions of the particles used for reconstruction of monomer1 (**c**) and monomer2 (**d**). Each cylinder represents one view, and the height of the cylinder is proportional to the number of particles for that view. **e, f,** Local resolution maps of monomer1 (**e**) and monomer2 (**f**).

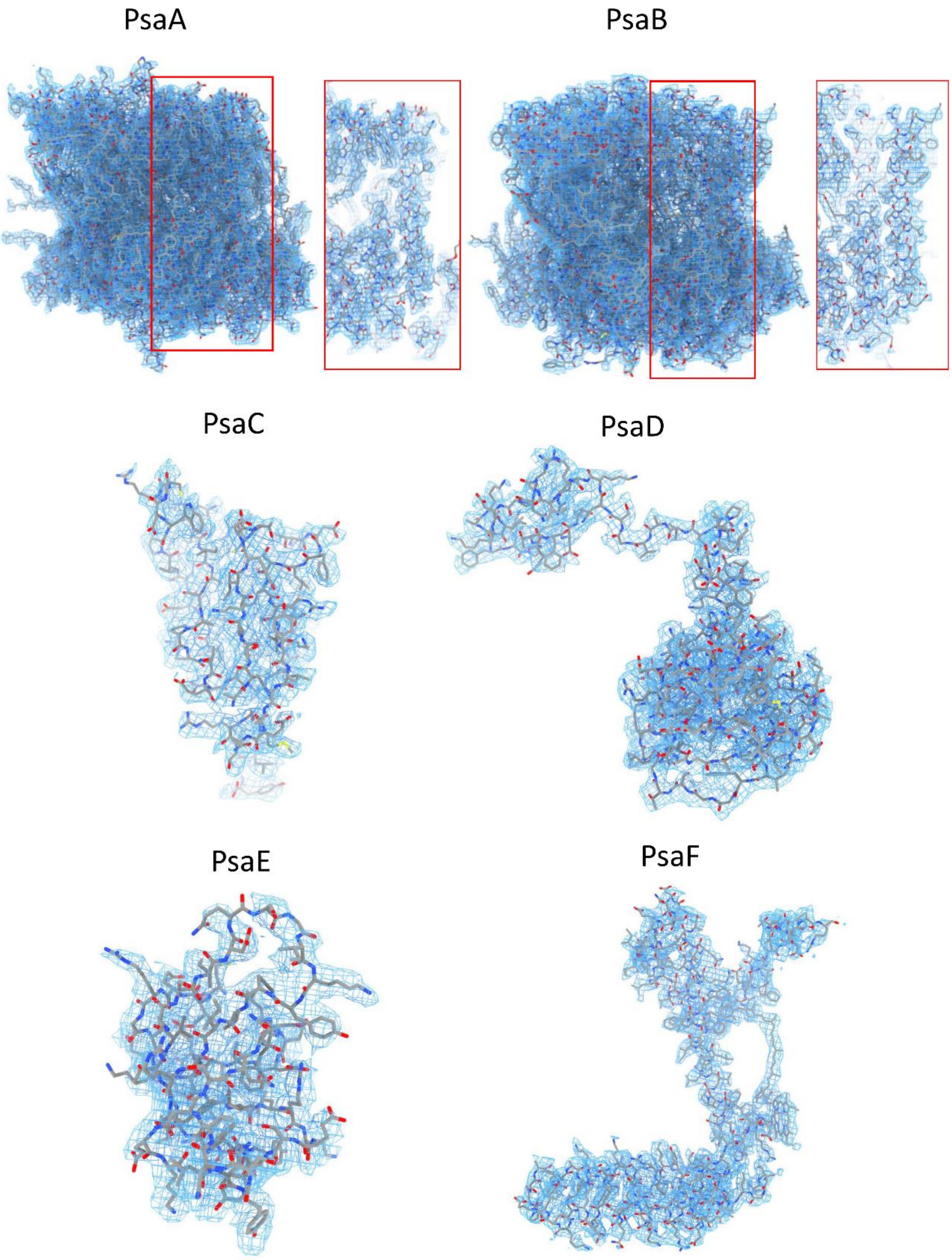
PsaA
PsaB
PsaC
PsaD
PsaE
PsaF

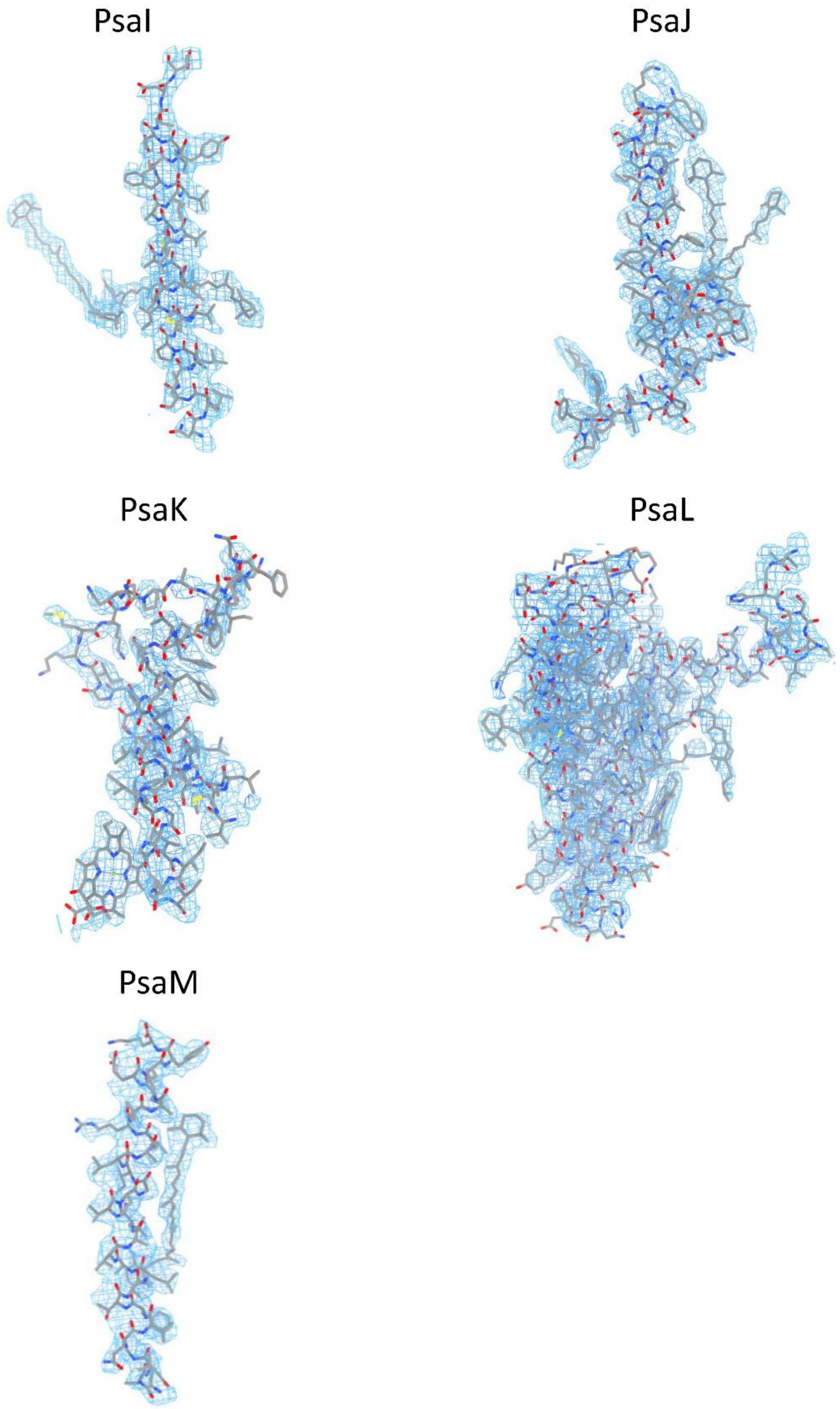

Supplementary Fig. 4. Cryo-EM density maps and structures of the PSI-monomer1 subunits. The densities for each subunit of PSI are shown as blue meshes, and the corresponding models are shown as gray sticks. Red boxes indicate the enlarged views for a part of the individual PsaA and PsaB subunits.

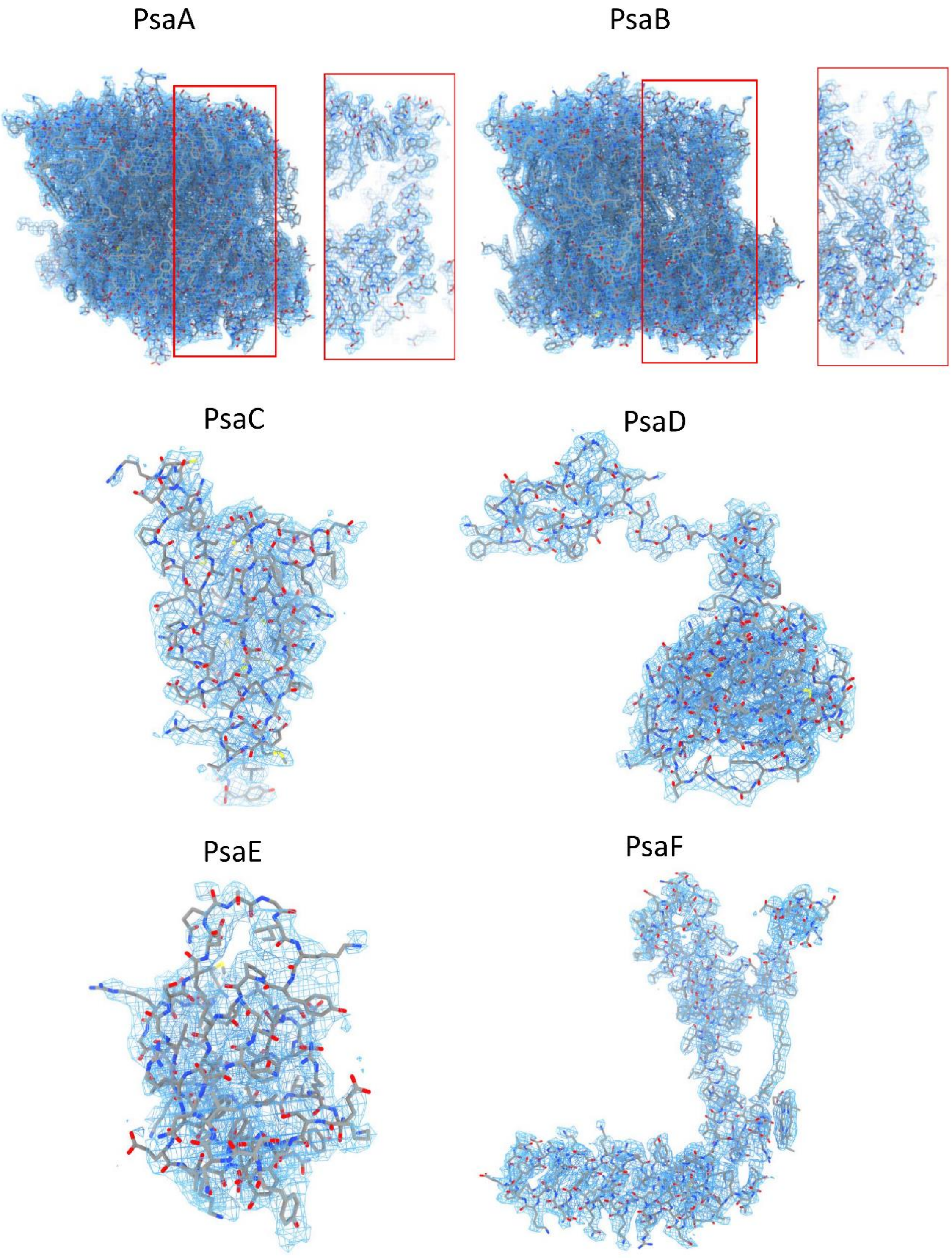
PsaA
PsaB
PsaC
PsaD
PsaE
PsaF

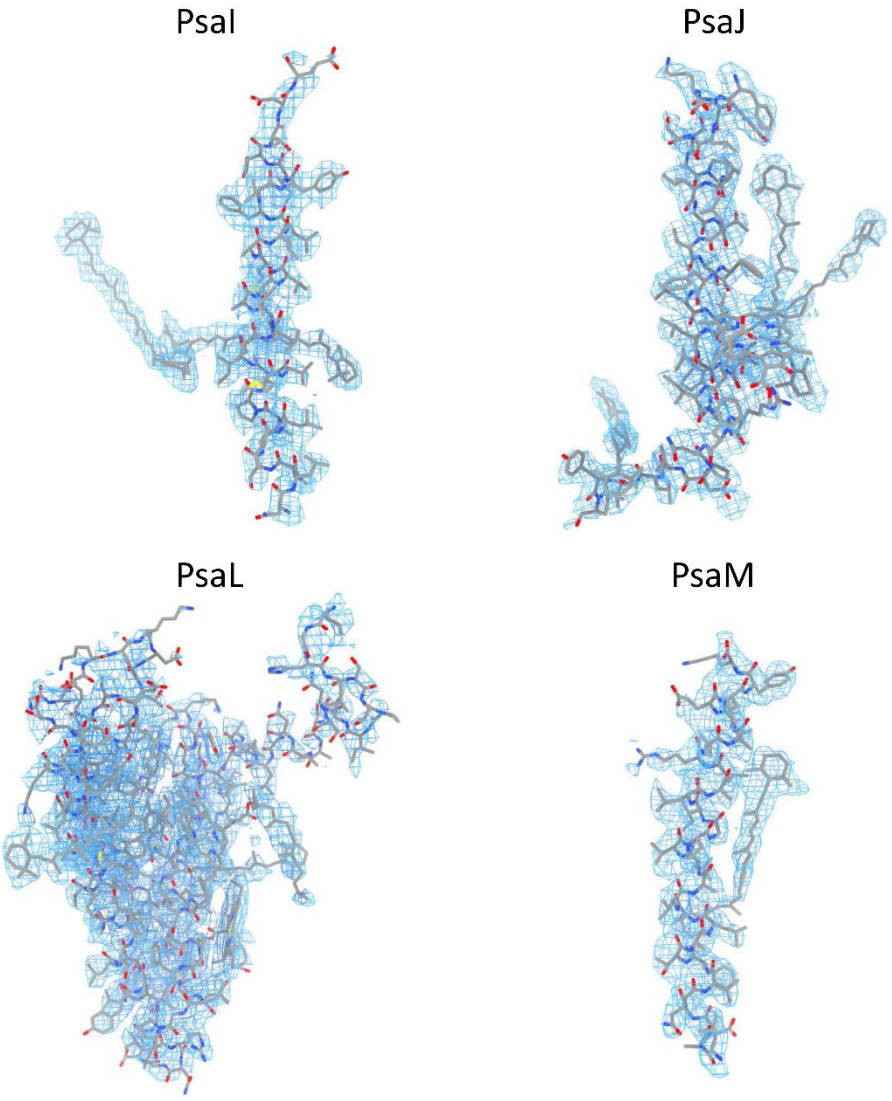

Supplementary Fig. 5. Cryo-EM density maps and structures of the PSI-monomer2 subunits. The densities for each subunit of PSI are shown as blue meshes, and the corresponding models are shown as gray sticks. Red boxes indicate the enlarged views for a part of the individual PsaA and PsaB subunits.

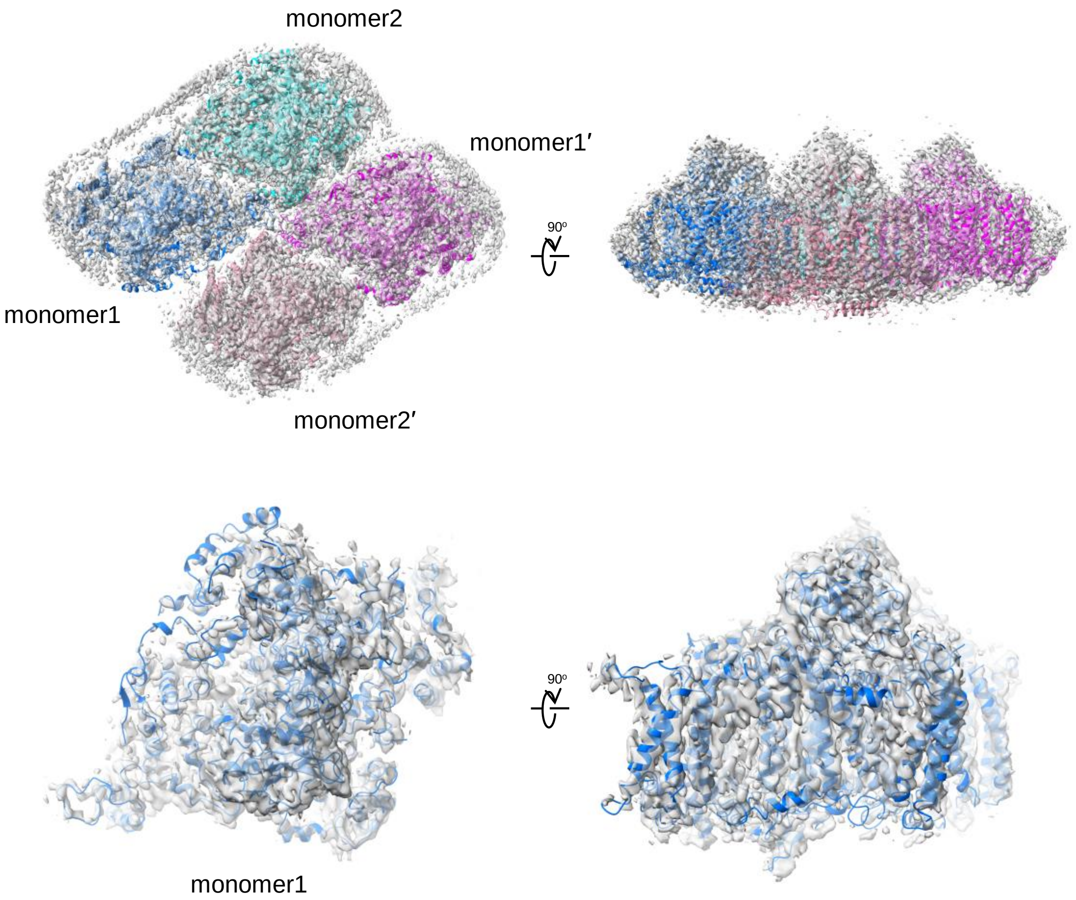

Supplementary Fig. 6. Overall structure of the wild-type PSI tetramer. The 3D cryo-EM density maps of the PSI tetramer superimposed with a cartoon model of the PSI tetramer, with a view along the membrane normal from the stromal side (upper left) and its side view (upper right). Lower panel shows the superimposition of the monomer1 structure with its cryo-EM density.

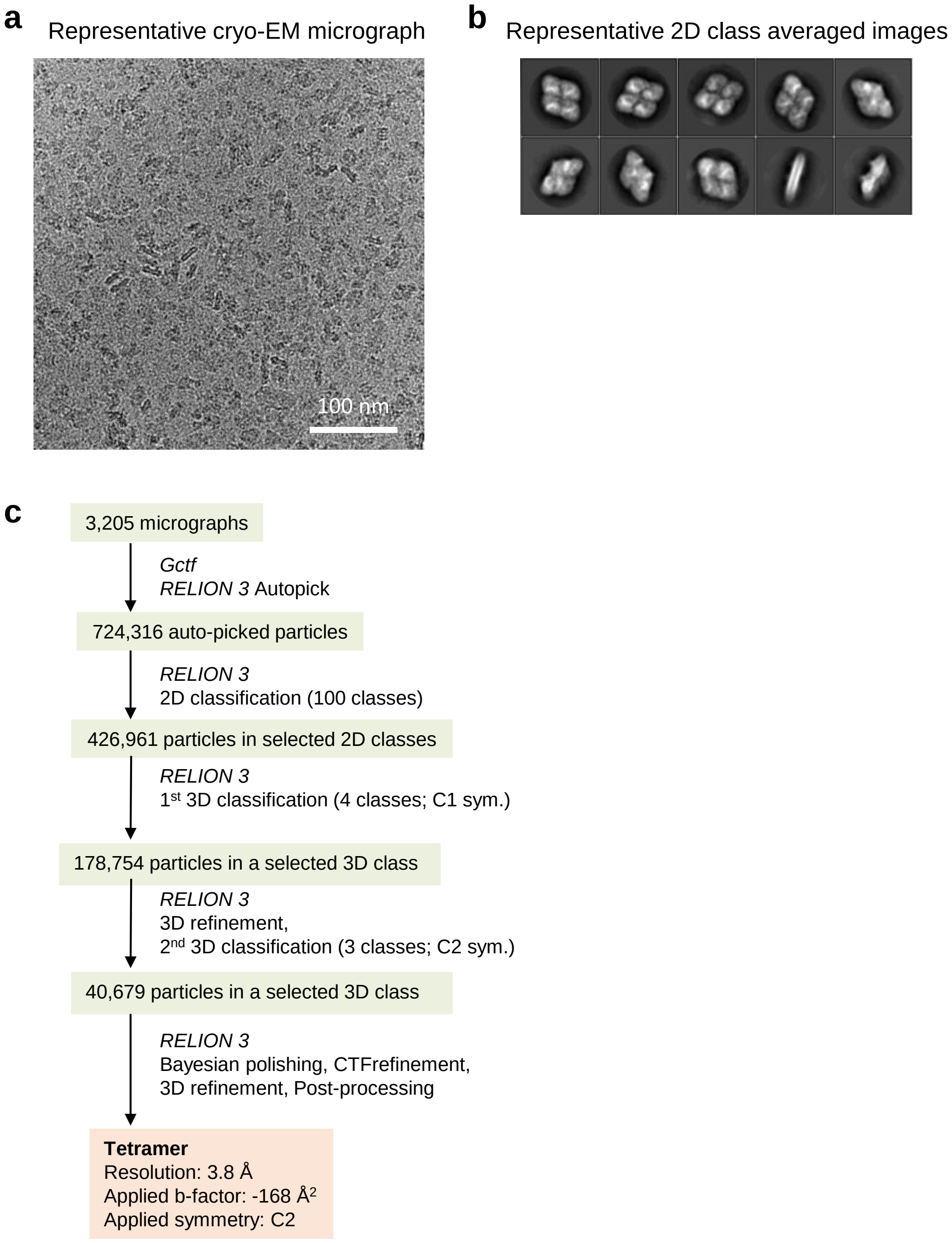

Supplementary Fig. 7. Cryo-EM data collection and processing of the GraFix PSI tetramer. a, Representative cryo-EM micrograph of the PSI tetramer. **b,** Representative 2D classes of the PSI tetramer. The box size is 437 Å. **c,** Schematic flowchart showing the classification scheme and data processing for the PSI tetramer. The overall PSI tetramer structure was reconstructed at 3.8 Å resolution from 40,679 particles. See Methods section for more details.

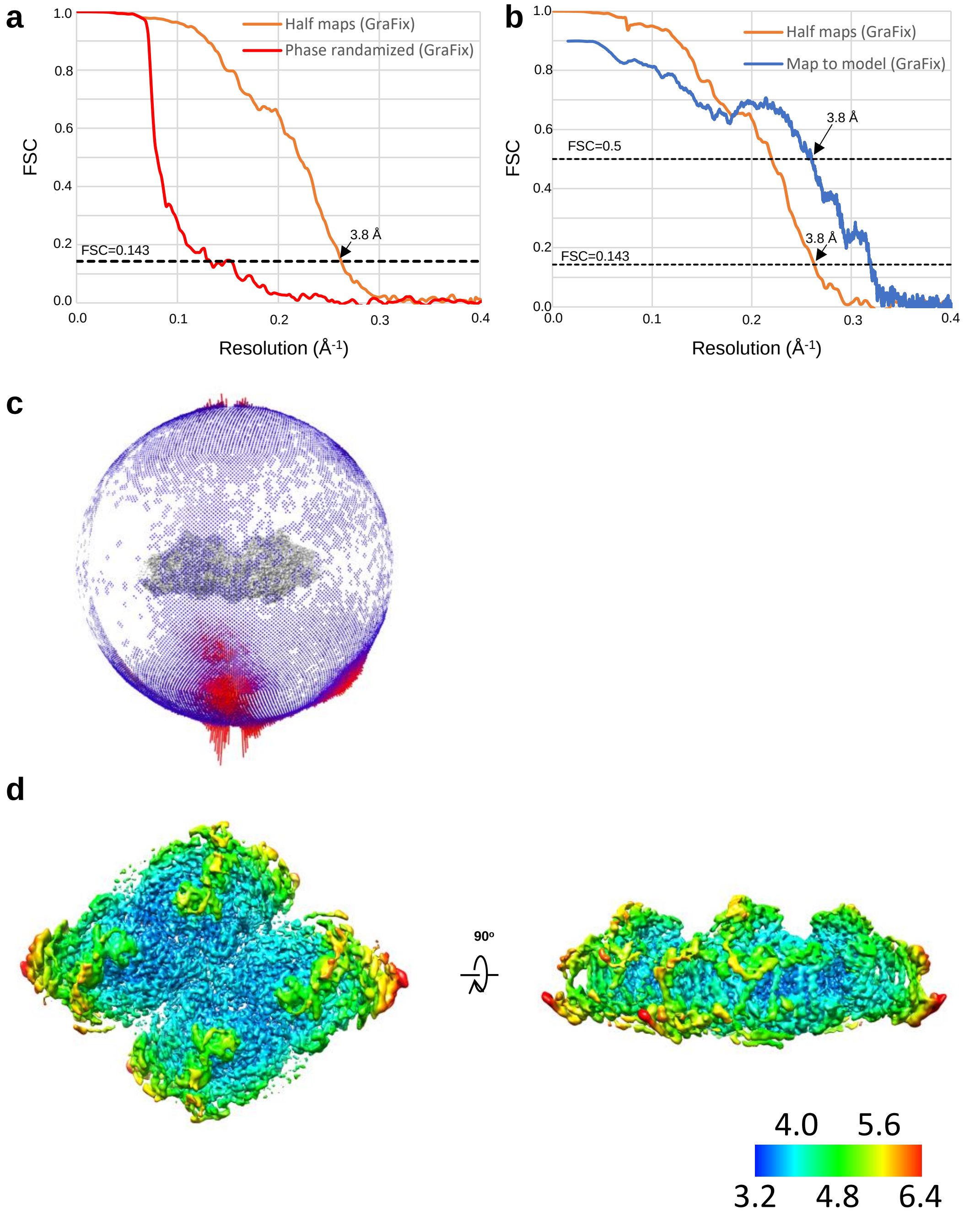

Supplementary Fig. 8. Evaluation of the cryo-EM map quality. a, FSC curves of the GraFix PSI tetramer for independently refined half-maps (half-maps) and phase randomized maps. **b,** FSC curves of the GraFix PSI tetramer for independently refined half-maps (half-maps) and full map vs. model (map to model). **c,** Angular distribution of the particles used for reconstruction of the GraFix PSI tetramer. Each cylinder represents one view, and the height of the cylinder is proportional to the number of particles for that view. **d,** Local resolution map of the GraFix PSI tetramer.

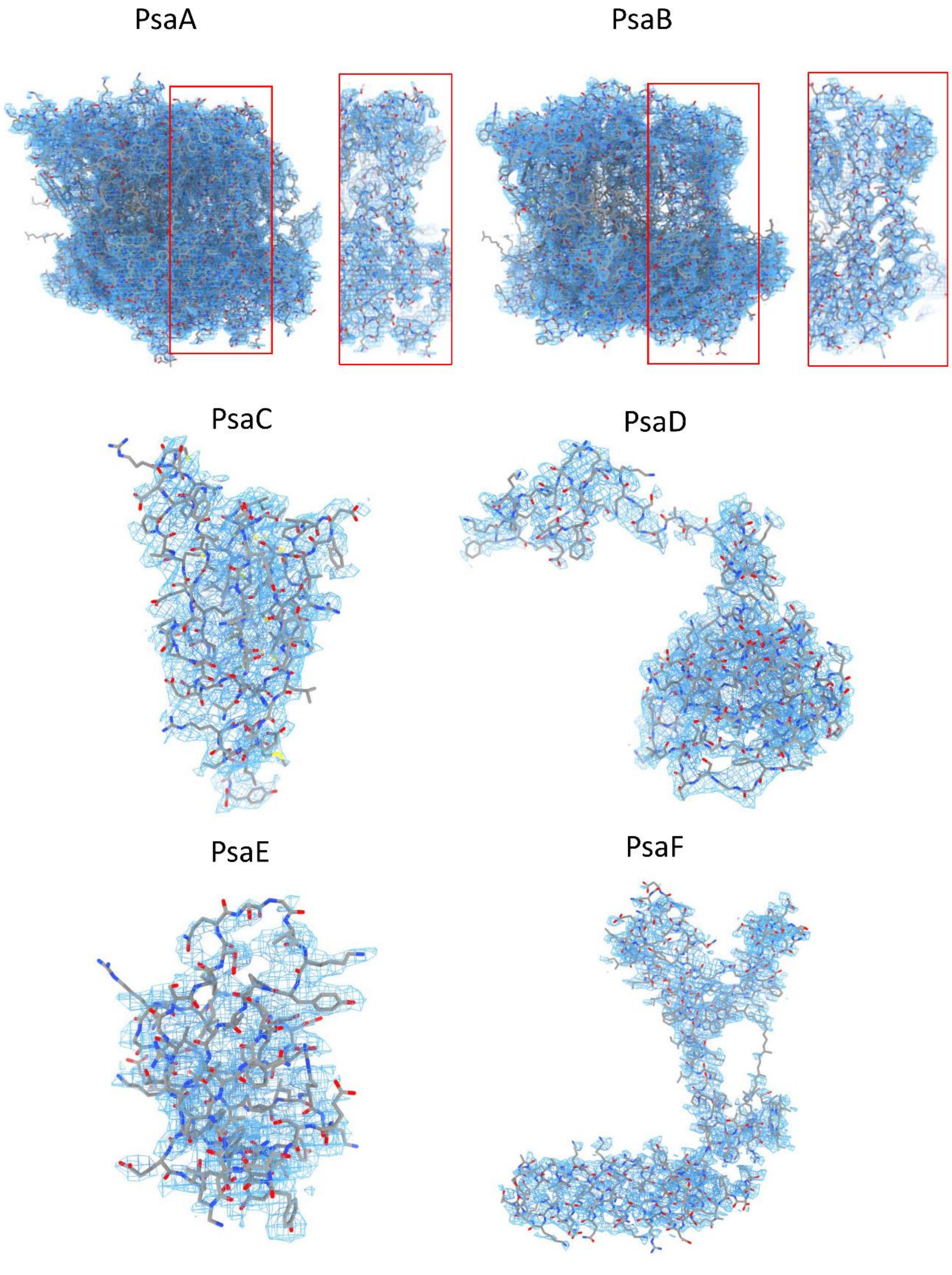
PsaA
PsaB
PsaC
PsaD
PsaE
PsaF

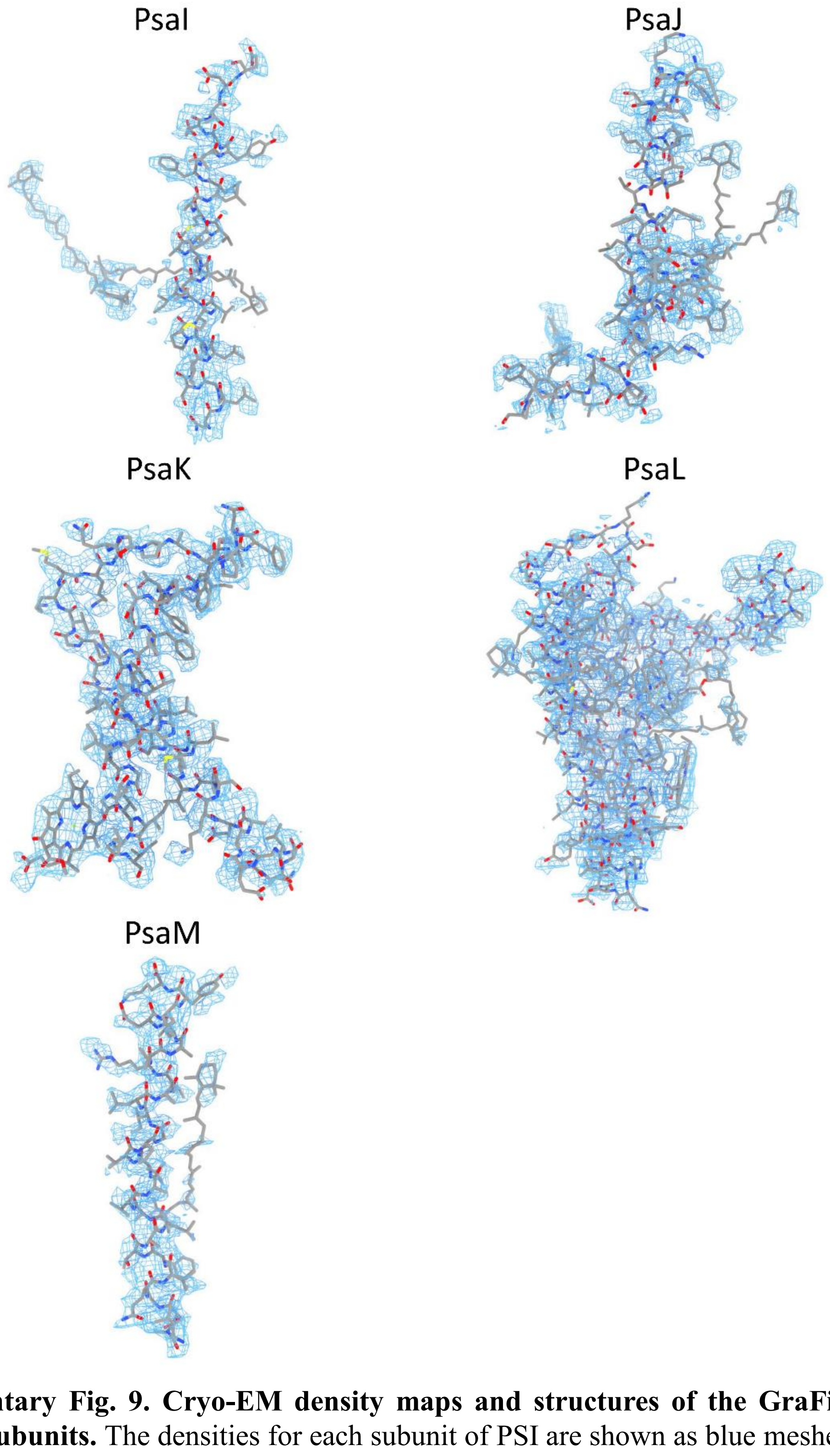

Supplementary Fig. 9. Cryo-EM density maps and structures of the GraFix PSI tetramer subunits. The densities for each subunit of PSI are shown as blue meshes, and the corresponding models are shown as gray sticks. Red boxes indicate the enlarged views for a part of the individual PsaA and PsaB subunits.

a

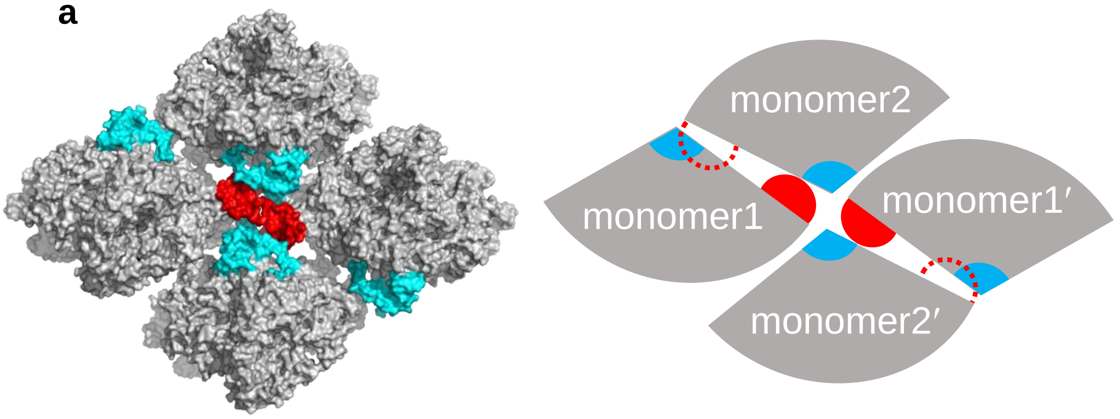

Cyanophora PSI tetramer (this study)

b

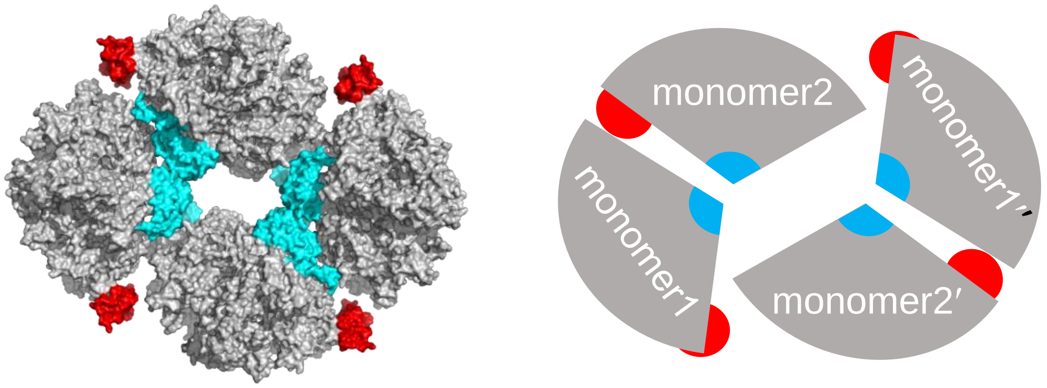

Anabaena PSI tetramer (6JEO)

Supplementary Fig. 10. Comparison of the organization patterns of the PSI tetramer between *Cyanophora* and *Anabaena*. a, *Cyanophora* PSI tetramer. **b,** *Anabaena* PSI tetramer (PDB: 6JEO). Red and cyan represent PsaK and PsaL, respectively. The areas circled by red dashed lines in the right side of panel **a** represent the sites of PsaK that are in conflict with the adjacent monomer and lost in the structure.

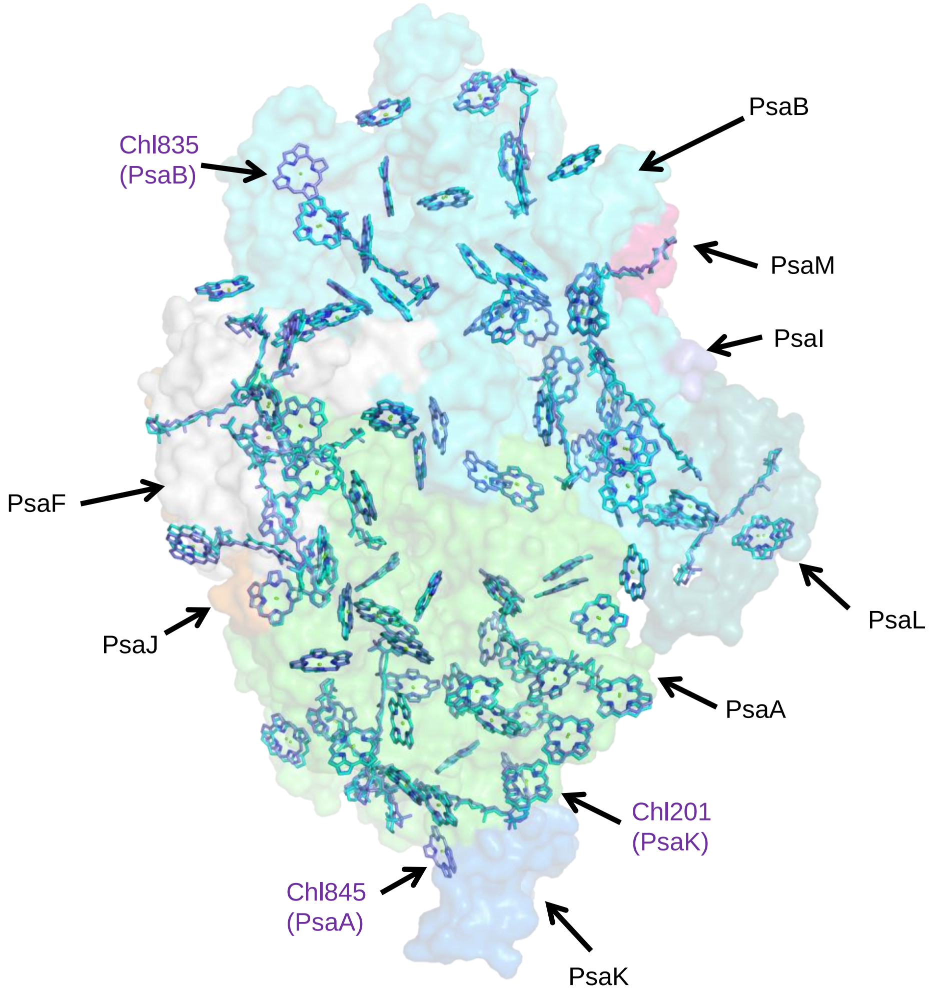

Supplementary Fig. 11. Superposition of the pigment molecules of monomer1 with those of monomer2. Arrangement of the pigments in monomer1 (purple) and monomer2 (cyan), with protein surface model viewed along the membrane normal from the stromal side. The stromal side extrinsic subunits PsaC, PsaD, and PsaE are deleted in the model for clarity.

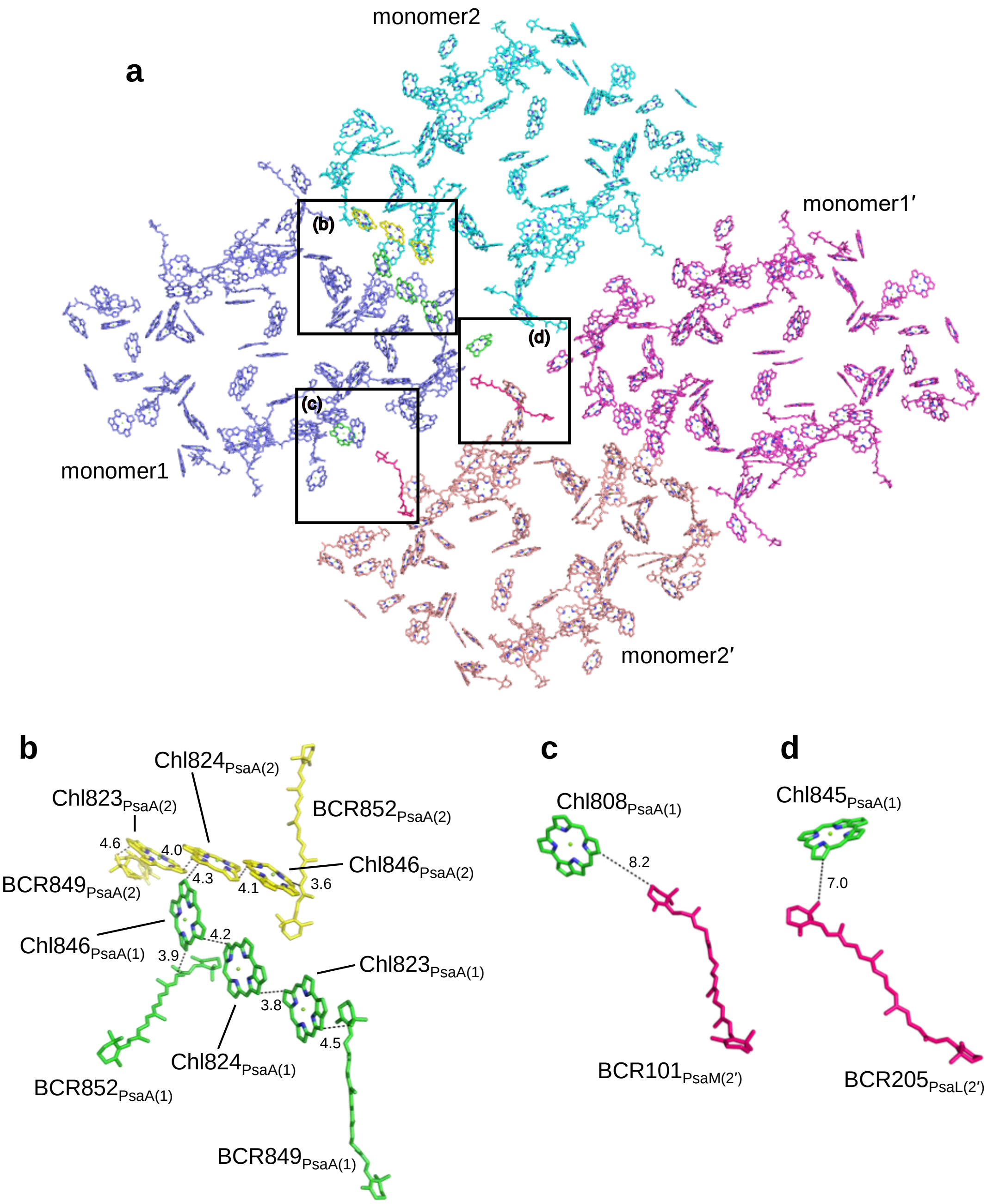

Supplementary Fig. 12. Arrangement of pigments in the *Cyanophora* PSI tetramer and their interactions. a, Arrangement of the pigments in the PSI tetramer viewed along the membrane normal from the stromal side. Pigments of monomer1, monomer2, monomer1′, and monomer2′ are colored in blue, cyan, magenta, and pink, respectively. A triply stacked Chl cluster in monomer1, and a triple Chl cluster in monomer2 that interacts with monomer1, are colored in green and yellow, respectively. Boxed areas are enlarged in the following panels. **b,** Interactions of pigments between monomer1 (green) and monomer2 (yellow) shown in box (**b**) of panel **a**. **c, d,** Interactions of two Chls in monomer1 and two *β*-carotenes in monomer2′ shown in boxes (**c**) and (**d**) of panel **a**.

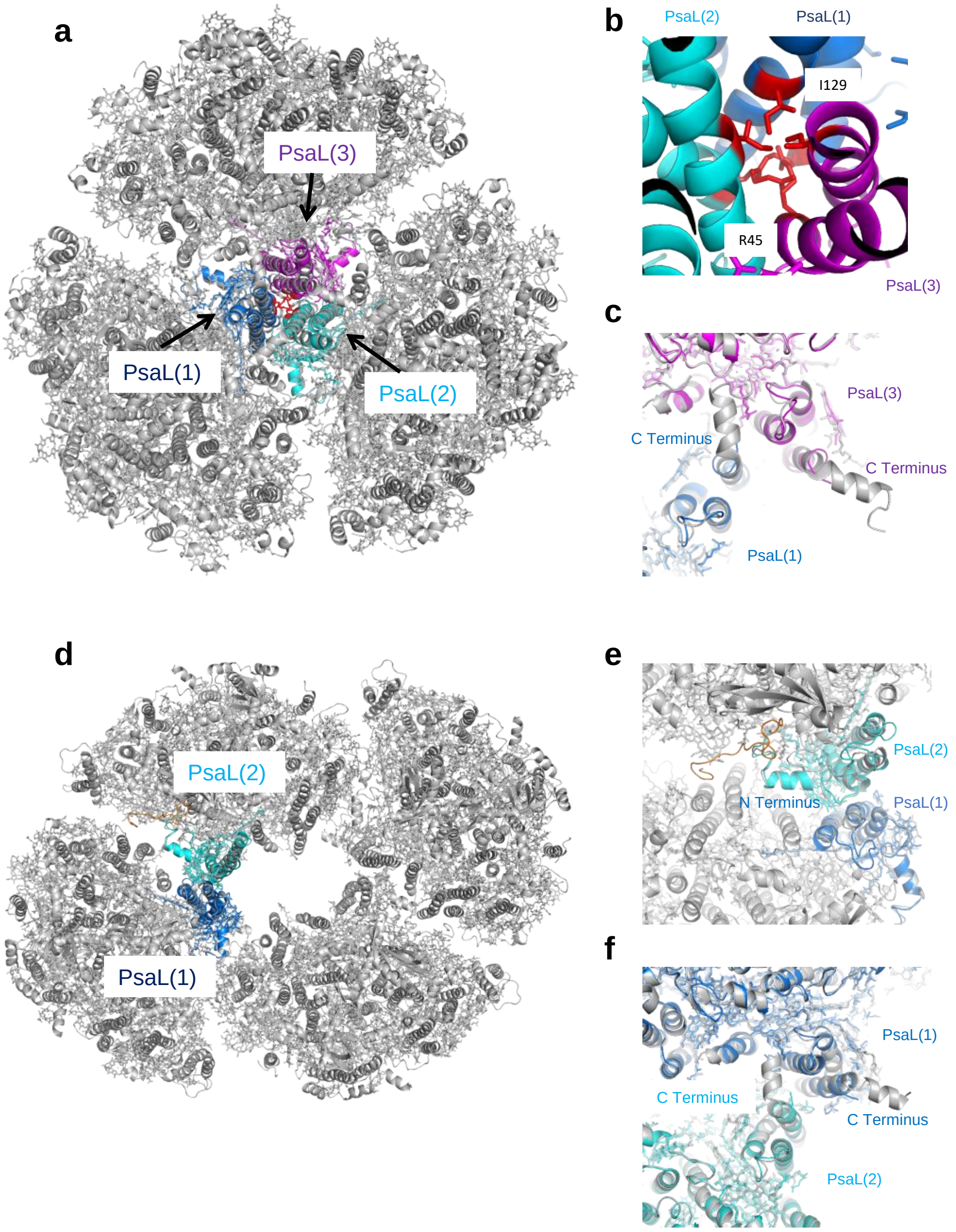

Supplementary Fig. 13. Structural modeling of *Cyanophora* PSI trimer and tetramer. a, Superposition of *Cyanophora* PSI monomers with the trimeric structure of PSI from *T. elongatus* (PDB: 1JB0), viewed along the membrane normal from the stromal side. The region having steric hindrances is colored in red. PsaL subunits in the three monomers labeled as 1–3 are depicted in blue, cyan, and purple, respectively. **b,** Close-up view of the steric hindrance of PsaL from the lumenal side. **c,** Close-up view of the C-terminus of PsaL from the lumenal side. **d,** Superposition of *Cyanophora* PSI monomers with the tetrameric structure of PSI from *Anabaena* sp. PCC 7120 (PDB: 6JEO), viewed along the membrane normal from the stromal side. **e,** Close-up view of the N-terminus of PsaL from the stromal side. A loop structure (orange) stands for the N-terminal loop of PsaL in *Anabaena* sp. PCC 7120. **f,** Close-up view of the C-terminus of PsaL from the lumenal side.

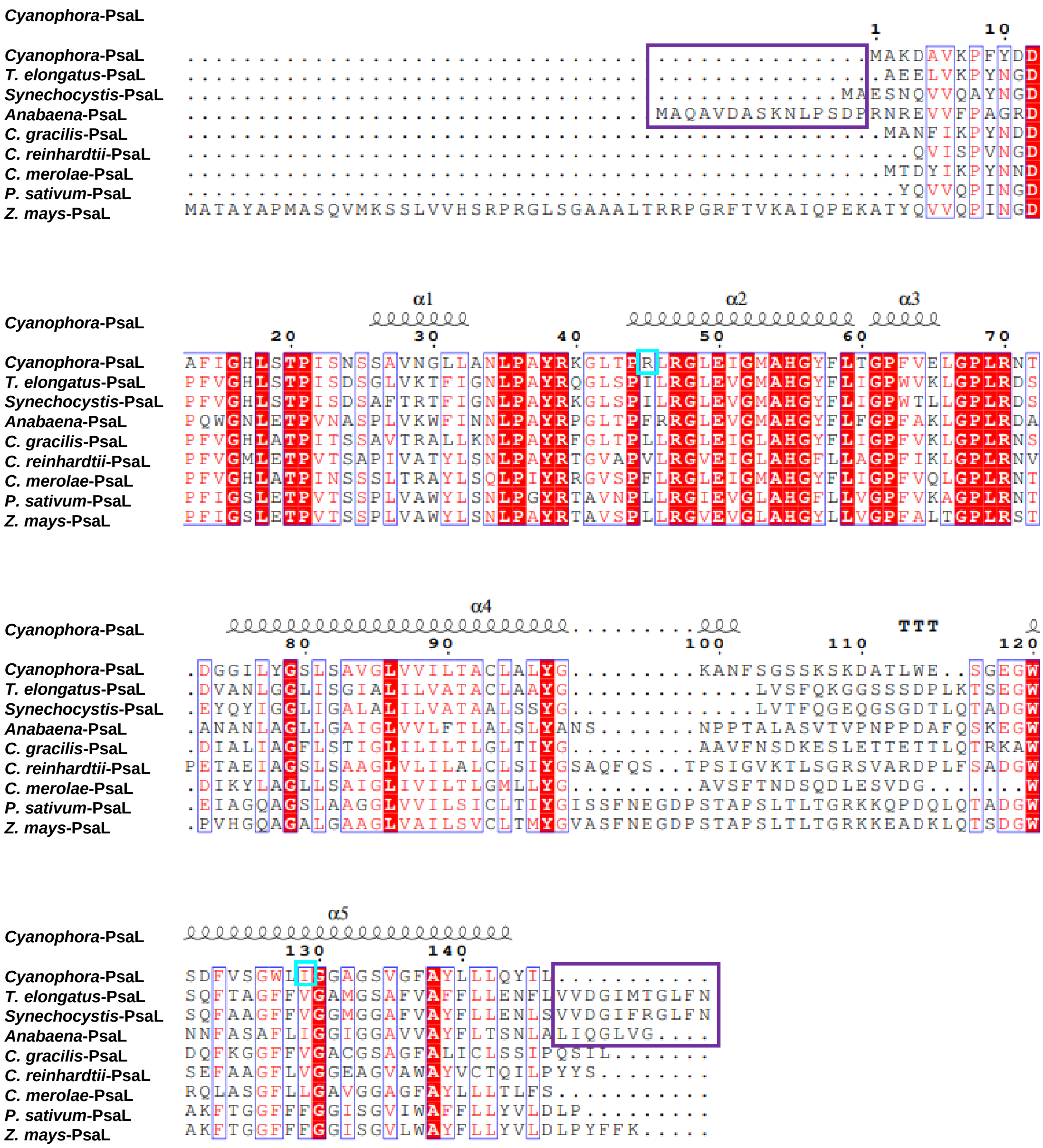

Supplementary Fig. 14. Multiple sequence alignment (CLUSTALW and ESPript) of PsaL among *Cyanophora* and other photosynthetic organisms. The amino acid residues of *Cyanophora* PsaL that cause steric hindrance with the cyanobacterial trimer are highlighted in cyan. Completely conserved residues are highlighted in red. The purple boxes show regions at the N-terminus and C-terminus that are lacking in *Cyanophora*. The species shown are *Cyanophora paradoxa*, *Thermosynechococcus elongatus* BP-1, *Synechocystis* sp. PCC 6803, *Anabaena* sp. PCC 7120, *Chaetoceros gracilis*, *Chlamydomonas reinhardtii*, *Cyanidioschyzon merolae*, *Pisum sativum*, and *Zea mays*.

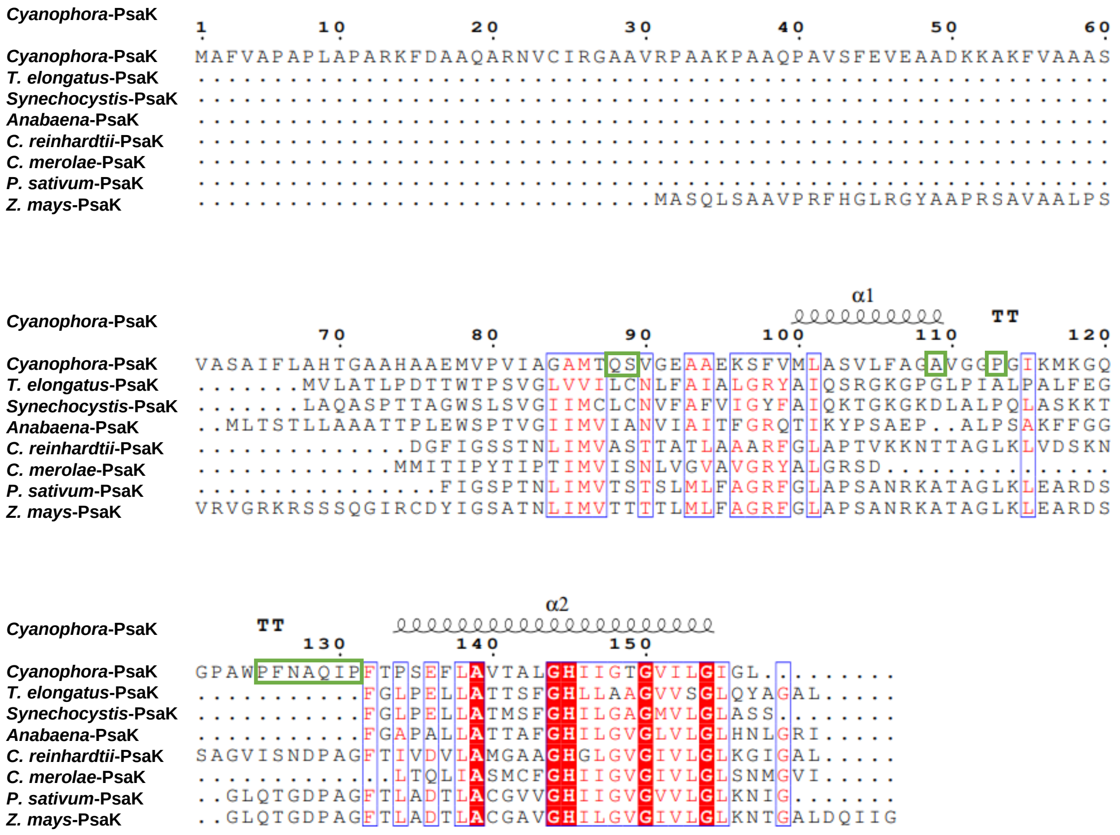

Supplementary Fig. 15. Multiple sequence alignment (CLUSTALW and ESPript) of PsaK among *Cyanophora* and other photosynthetic organisms. The amino acid residues in the contact regions at the center of the PSI tetramer are highlighted in green. Completely conserved residues are highlighted in red. The species shown are *Cyanophora paradoxa*, *Thermosynechococcus elongatus* BP-1, *Synechocystis* sp. PCC 6803, *Anabaena* sp. PCC 7120, *Chlamydomonas reinhardtii*, *Cyanidioschyzon merolae*, *Pisum sativum*, and *Zea mays*.

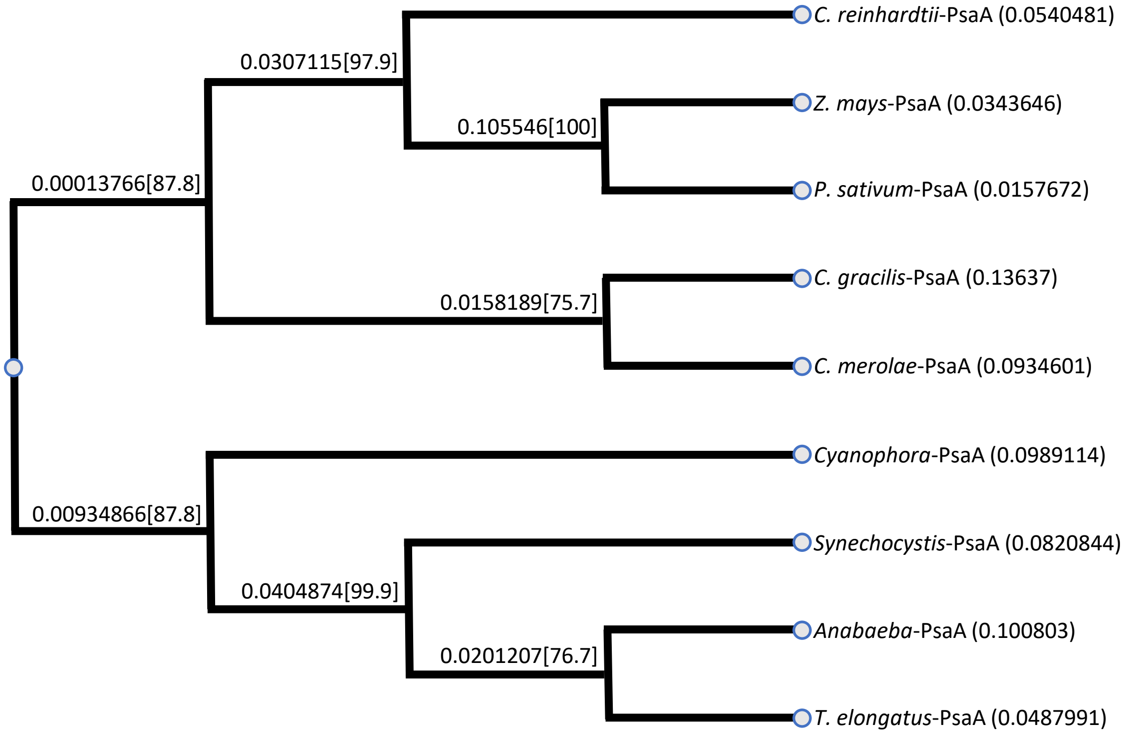

Supplementary Fig. 16. Phylogenetic analysis of the PsaA proteins among *Cyanophora* and other photosynthetic organisms. The species shown are *Cyanophora paradoxa*, *Thermosynechococcus elongatus* BP-1, *Synechocystis* sp. PCC 6803, *Anabaena* sp. PCC 7120, *Chaetoceros gracilis*, *Chlamydomonas reinhardtii*, *Cyanidioschyzon merolae*, *Pisum sativum*, and *Zea mays*.

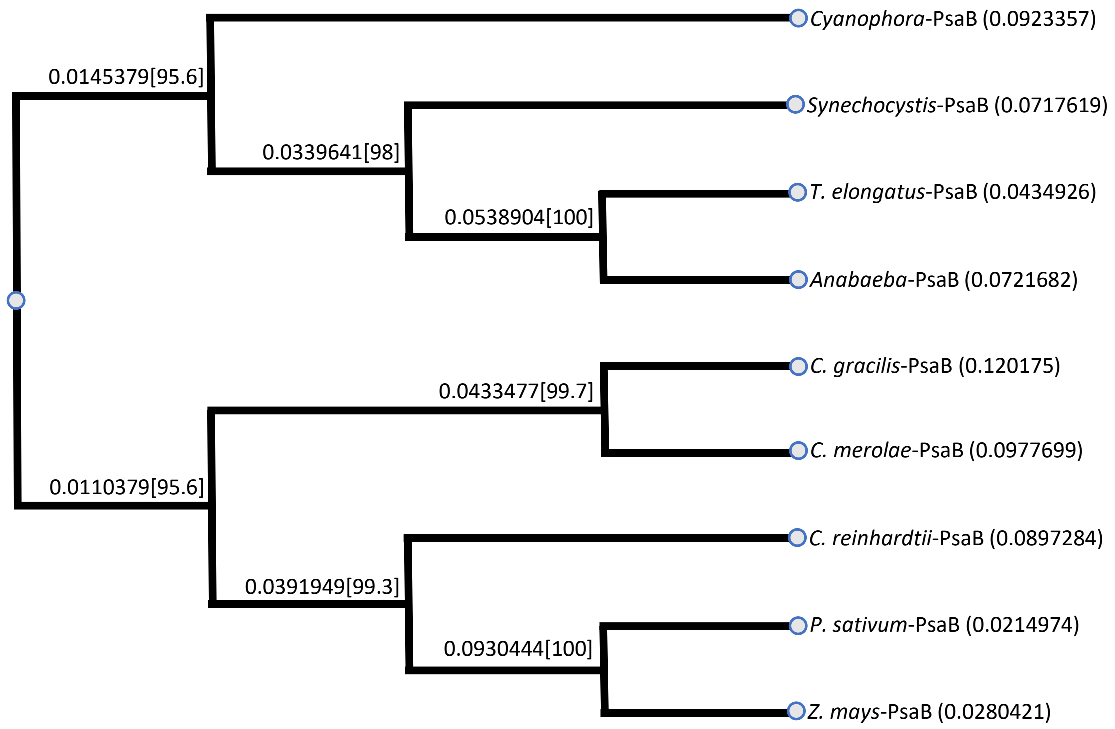

Supplementary Fig. 17. Phylogenetic analysis of the PsaB proteins among *Cyanophora* and other photosynthetic organisms. The species shown are *Cyanophora paradoxa*, *Thermosynechococcus elongatus* BP-1, *Synechocystis* sp. PCC 6803, *Anabaena* sp. PCC 7120, *Chaetoceros gracilis*, *Chlamydomonas reinhardtii*, *Cyanidioschyzon merolae*, *Pisum sativum*, and *Zea mays*.

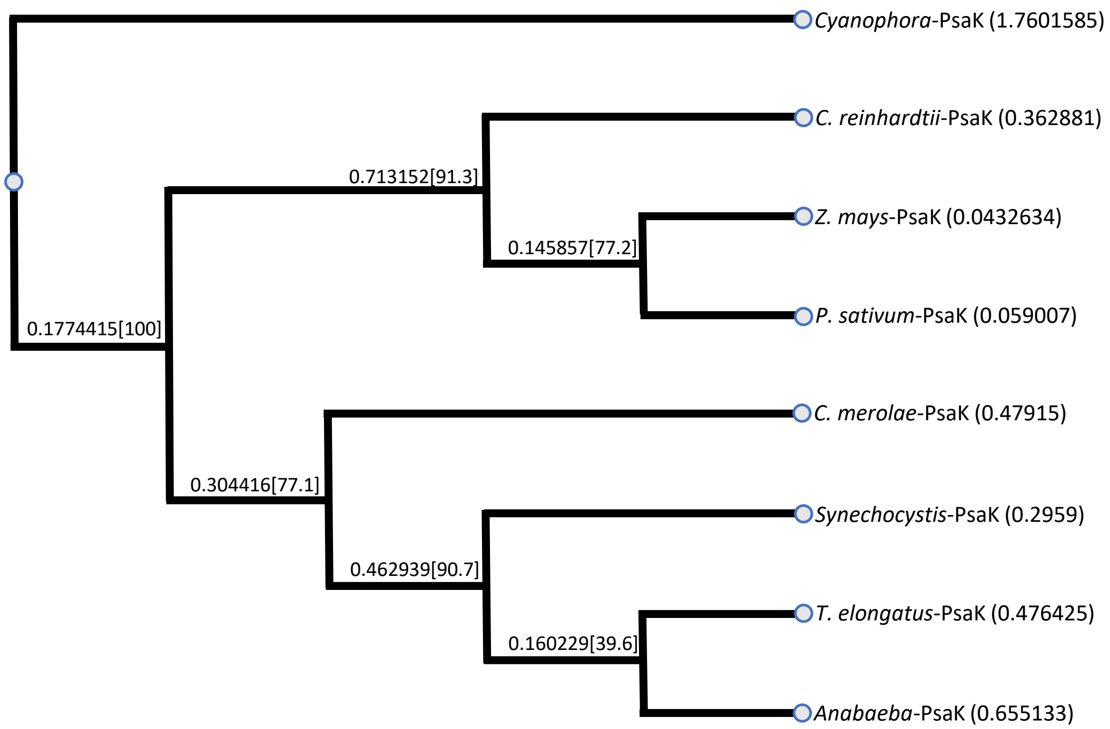

Supplementary Fig. 18. Phylogenetic analysis of the PsaK proteins among *Cyanophora* and other photosynthetic organisms. The species shown are *Cyanophora paradoxa*, *Thermosynechococcus elongatus* BP-1, *Synechocystis* sp. PCC 6803, *Anabaena* sp. PCC 7120, *Chlamydomonas reinhardtii*, *Cyanidioschyzon merolae*, *Pisum sativum*, and *Zea mays*.

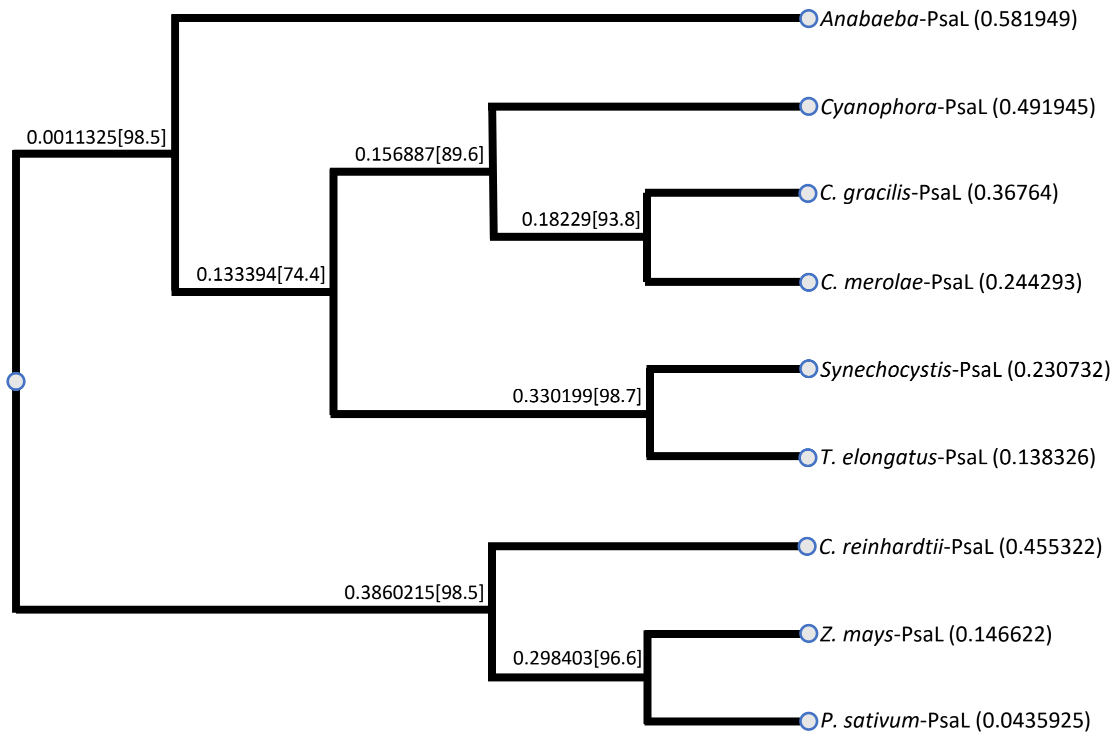

Supplementary Fig. 19. Phylogenetic analysis of the PsaL proteins among *Cyanophora* and other photosynthetic organisms. The species shown are *Cyanophora paradoxa*, *Thermosynechococcus elongatus* BP-1, *Synechocystis* sp. PCC 6803, *Anabaena* sp. PCC 7120, *Chaetoceros gracilis*, *Chlamydomonas reinhardtii*, *Cyanidioschyzon merolae*, *Pisum sativum*, and *Zea mays*.

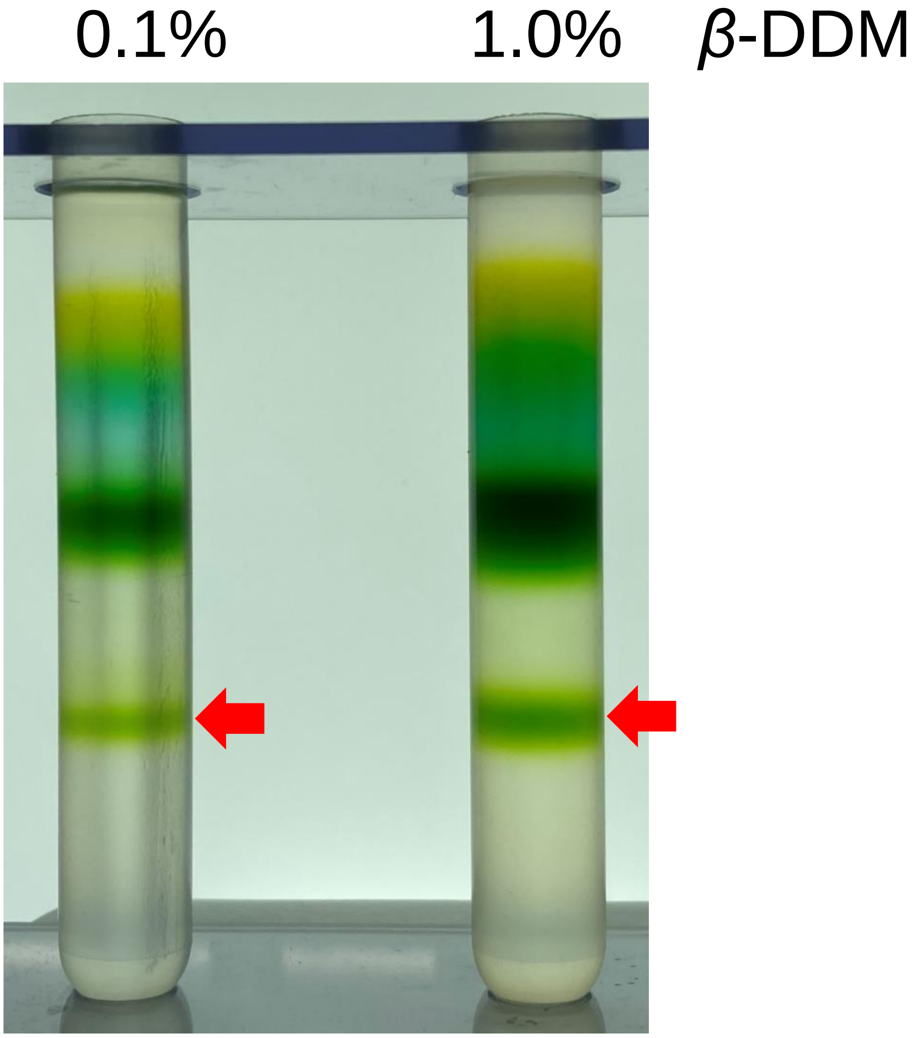

Supplementary Fig. 20. Trehalose gradient centrifugation after solubilizing the thylakoids with 0.1% or 1.0% *β*-DDM. Red arrows indicate the PSI tetramer. Thylakoid membranes were solubilized with either 0.1% (left) or 1% *β*-DDM (right) at a Chl concentration of 0.25 mg mL^{-1} for 30 min on ice in the dark with gentle stirring. After centrifugation at 20,000 × g for 10 min at 4°C, the resultant supernatant were concentrated using a 100 kDa cut-off filter (Amicon Ultra; Millipore) at 4,000 × g, and then the concentrated samples were loaded onto a linear trehalose gradient of 10–40% (w/v) in a medium containing 20 mM Mes-NaOH (pH 6.5), 0.2 M NaCl, and 0.1% *β*-DDM. The PSI-tetramer fraction was obtained after centrifugation at 154,000 × g for 18 h at 4°C. Data is representative of three independent experiments.

Supplementary Table 1. Statistics of data collection, processing, and refinement.

Complex	Wild-type PSI monomer1	Wild-type PSI monomer2	Wild-type PSI tetramer	GraFix PSI tetramer
PDB ID	7DR0	7DR1	-	7DR2
EMDB ID	EMD-30820	EMD-30821	EMD-30822	EMD-30823
Data collection and processing				
Microscope		Talos Arctica		
Detector		Falcon 3EC direct electron detector		
Magnification		92000		
Voltage (kV)		200		
Defocus range (μm)		-2.0 to -4.0		-1.5 to -3.5
Pixel size (Å)		1.093		
Total electron dose (e^-/Å^2)		50		
Exposure time (s)		3.7 and 4.0		3.2
Number of frames per image		37 and 40		32
Number of micrographs		4,515		3,205
Initial particle images (no.)		1,603,082		724,316
Final particle images (no.)	70,920	110,380	145,567	40,679
Map resolution (Å)	3.3	3.2	4.0	3.8
Applied b-factor (Å^2)	-107	-117	-216	-168
Applied symmetry	C1	C1	C2	C2
Refinement				
Initial model used	Homology model	Homology model	-	Homology model
Model resolution (Å)	3.3	3.2	-	3.8
FSC threshold	0.5	0.5	-	0.5
No. of atoms				
Protein	16,975	16,572	-	67,224
Ligand	5,771	5,606	-	22,732
B factors (Å^2)				
Protein	44.0	35.0	-	69.5
Ligand	34.3	26.2	-	66.3
R.m.s deviations				
Bond lengths (Å)	0.009	0.007	-	0.009
Bond angles (°)	1.50	1.53	-	1.67
Validation				
MolProbity score	1.91	1.84	-	2.12
Clashscore	9.16	8.09	-	11.5
Poor rotamers (%)	0.62	0.75	-	1.27
EMRinger score	3.4	3.6	-	1.58
Ramachandran plot				
Favored (%)	93.62	93.98	-	92.58
Allowed (%)	6.15	5.74	-	7.16
Disallowed (%)	0.23	0.29	-	0.26

Supplementary Table 2. Cofactors in each monomer unit of the *Cyanophora* PSI tetramer and comparison with a monomer of the *T. elongatus* PSI trimer.

Protein		Chlorophyll	Carotenoid	Lipid	Others
PsaA	monomer1	46 Chl *a*	6 BCR	2 LHG	1 [4Fe-4S] cluster,<br>1 phylloquinone
	monomer2	45 Chl *a*	6 BCR	2 LHG	1 [4Fe-4S] cluster,<br>1 phylloquinone
	T. elongatus PSI[†]	46 Chl *a*	6 BCR	2 LHG	1 [4Fe-4S] cluster,<br>1 phylloquinone
PsaB	monomer1	32 Chl *a*	4 BCR	1 LMG	1 phylloquinone
	monomer2	31 Chl *a*	4 BCR	1 LMG	1 phylloquinone
	T. elongatus PSI[†]	41 Chl *a*	7 BCR	1 LMG<br>1 LHG	1 phylloquinone
PsaC	monomer1	-	-	-	2 [4Fe-4S] cluster
	monomer2	-	-	-	2 [4Fe-4S] cluster
	T. elongatus PSI[†]	-	-	-	2 [4Fe-4S] cluster
PsaD	monomer1	-	-	-	-
	monomer2	-	-	-	-
	T. elongatus PSI[†]	-	-	-	-
PsaE	monomer1	-	-	-	-
	monomer2	-	-	-	-
	T. elongatus PSI[†]	-	-	-	-
PsaF	monomer1	1 Chl *a*	1 BCR	-	-
	monomer2	1 Chl *a*	1 BCR	-	-
	T. elongatus PSI[†]	1 Chl *a*	1 BCR	-	-
PsaI	monomer1	-	2 BCR	-	-
	monomer2	-	2 BCR	-	-
	T. elongatus PSI[†]	-	2 BCR	-	-
PsaJ	monomer1	1 Chl *a*	3 BCR	-	-
	monomer2	1 Chl *a*	3 BCR	-	-
	T. elongatus PSI[†]	2 Chl *a*	3 BCR	-	-
PsaK[⁋]	monomer1	1 Chl *a*	-	-	-
	monomer2	-	-	-	-
	T. elongatus PSI[†]	1 Chl *a*	-	-	-
PsaL	monomer1	3 Chl *a*	2 BCR	-	-
	monomer2	3 Chl *a*	2 BCR	-	-
	T. elongatus PSI[†]	3 Chl *a*	2 BCR	-	-
PsaM	monomer1	-	1 BCR	-	-
	monomer2	-	1 BCR	-	-
	T. elongatus PSI[†]	1 Chl *a*	1 BCR	-	-
PsbX[⁋]	monomer1	-	-	-	-
	monomer2	-	-	-	-
	T. elongatus PSI[†]	1 Chl *a*	-	-	-
Total	monomer1	84	19	3	5
	monomer2	81	19	3	5
	T. elongatus PSI[†]	96	22	4	5

BCR, β-carotene; LMG, distearoylmonogalactosyl diglyceride; LHG, dipalmitoylphosphatidyl glycerol.

[†]PDB: 1JB0.

[⁋]PsaX in monomer1 and PsaK and PsaX in monomer2 are not found in the *Cyanophora* PSI.

Supplementary Table 3. Chls lost in the *Cyanophora* PSI tetramer and their ligands.

	T. elongatus PSI[†]		*Cyanophora*-monomer1		*Cyanophora*-monomer2	
	Chls[⁋]	Ligands	Chls[⁋]	Ligands	Chls[⁋]	Ligands
PsaA	1402	-	845	PsaK-H145	-	-
PsaB	1209	PsaB-H176	-	PsaB-H177[§]	-	PsaB-H177[§]
	1216	H_2O	-	-	-	-
	1217	PsaB-H288	-	PsaB-H289[§]	-	PsaB-H289[§]
	1218	PsaB-H298	-	PsaB-H299[§*]	-	PsaB-H299[§*]
	1219	H_2O	-	-	-	-
	1220	PsaB-H322	-	PsaB-H319[§*]	-	PsaB-H319[§*]
	1221	PsaB-Y329	-	PsaB-V326[§]	-	PsaB-V326[§]
	1227	PsaB-H417	-	PsaB-H414[§]	-	PsaB-H414[§]
	1232	H_2O	835	-	-	-
	1233	H_2O	-	-	-	-
PsaJ	1303	PsaJ-H39	-	PsaJ-Y38[§]	-	PsaJ-Y38[§]
PsaK	1401	-	201	-	-	-
PsaM	1601	H_2O	-	-	-	-
PsaX	1701	PsaX-N23	-	-	-	-

[†]PDB: 1JB0.
[⁋]These Chls listed were not found or only present in monomer1 in the *Cyanophora* PSI structure compared with the *T. elongatus* PSI structure.
[§]These amino acids in the *Cyanophora* PSI structure are located at the same positions as the amino acid ligands in the *T. elongatus* PSI structure, although they have no corresponding Chl molecules.
*Disordered.
